# Self-leadership of nurses in a critical care outreach service: The development of a conceptual framework

**DOI:** 10.4102/hsag.v27i0.1965

**Published:** 2022-07-29

**Authors:** Carine Prinsloo, Karien Jooste

**Affiliations:** 1 School of Nursing, Faculty of Community and Health Sciences, University of the Western Cape, Cape Town, South Africa; 2 Department of Health Studies, College of Human Sciences, University of South Africa, Pretoria, South Africa; 3 Department of Nursing Sciences, Faculty of Health and Wellness Sciences, Cape Peninsula University of Technology, Cape Town, South Africa

**Keywords:** leadership, self-leadership, critical care outreach service, conceptual framework, nurses

## Abstract

**Background:**

Globally, critical care outreach services (CCOS) were implemented in health care facilities; however, compliance with guidelines is poor. The authors have noticed that a gap exists in the literature on how self-leadership might influence nurses’ implementation of CCOS. Self-leadership is about leadership applied to oneself. Critical care outreach services assist nurses with the nursing care of a patient whose health is declining. Leadership is needed for the successful implementation of CCOS.

**Aim:**

This article aims to outline the method the authors followed for developing the conceptual framework for how self-leadership amongst nurses influenced the functioning of CCOS.

**Setting:**

The research was conducted at a private hospital in Pretoria.

**Methods:**

A qualitative approach was followed to provide an accurate description of nurses’ experiences on their self-leadership in a CCOS. The practice-oriented theory of Dickoff (1968) was the reasoning map for developing and constructing the conceptual framework.

**Results:**

Bedside nurses experienced the following self-leadership strategies: constructive thought patterns, natural rewards and behaviours focused on their implementation of CCOS.

**Conclusion:**

The conceptual framework was part of another study and provided the authors with a rationale that guided the authors with the development of self-leadership strategies in a CCOS.

**Contribution:**

The conceptual framework provided the authors with an understanding of how nurses’ self-leadership influenced the implementation of CCOS. The conceptual framework can also assist in developing training programmes for nurses to improve their self-leadership and ultimately improve nurses’ competence in providing quality nursing care to patients.

## Introduction and background

Literature examining the relationship between self-leadership and nursing practice (Gomes et al. 2015; Jooste & Cairns 2014; Kim & Kim 2019) has gained more attention in recent years; however, the authors have noticed a gap in the literature concerning how self-leadership might influence nurses’ implementation of critical care outreach service (CCOS).

Globally, several CCOS approaches were implemented in health care facilities; however, compliance with the implementation guidelines is poor (Credland, Dyson & Johnson 2018). Critical care outreach service is an approach where patients in low-acuity wards whose health stands a chance of declining or whose health is declining are identified by the bedside nurse and referred to the CCOS to receive personalised interventions from the CCOS and bedside nurses being mentored by the critical care outreach team (CCOT) (Wood et al. 2017). The assistance and mentoring of bedside nurses increased bedside nurses’ competence in providing nursing care to patients whose health is declining. Globally, diverse CCOS approaches exist, and Prinsloo (2020) stated that the diverse CCOS approaches all share common features such as the tracking of vital data by means of, for example, modified early warning score (MEWS) as a referral algorithm. Critical care outreach services assist nursing staff to undertake timely, suitable and personalised interventions when a patient is referred to the CCOS. In a private hospital (research setting), the CCOS is nurse-led. Bedside nurses calculate patients’ MEWS after each vital data monitoring and must refer patients to the CCOS when the guidelines indicate it. Jeddian et al. (2017) reported that nurses felt CCOS unnecessarily added to their amount of work and hampered their nursing care routines.

Self-leadership is a process through which persons influence their own behaviour by applying three sets of approaches, namely behaviour-focused strategies, constructive thought patterns and natural reward strategies (Neck & Houghton, 2006, Neck, Manz & Houghton 2020).

Behaviour-focused strategies assist people to manage their own behaviour and consist of self-imposed approaches such as self-observation, self-goal setting, self-cueing, self-reward and self-correcting feedback (Neck et al. 2020). To change ineffective behaviour, one needs to be aware of the ineffective behaviour; therefore, self-observation can be seen as the first step in changing ineffective behaviour. An example of self-observation is when a nurse determines that he or she lacks knowledge on the application of MEWS in the CCOS and sets himself accepted herself the goal (goal-setting) to get information on the application of MEWS in CCOS. This improves nurses’ competence in providing quality nursing care to patients whose health is declining. Including a poster on calculating and application of MEWS in patients files is an example of self-cueing. Self-reward, for example, can be as simple as congratulating oneself on achieving one’s goal, or it may be more concrete, such as acquiring something one wants. Self-correcting feedback can be seen, for example, when one reflects and thinks about what one need to do to get what is wanted or needed.

Constructive thought patterns reform perceptual processes to enable more positive thought patterns that may enhance performance, which contains the disregarding of irrational or pessimistic self-talk in favour of more useful and positive self-talk (Neck & Houghton 2006, Neck et al. 2020). Another constructive thought pattern approach is mental imagery, which involves visualising the successful execution of a task in advance of the actual task (Neck et al. 2020). The nurse, for example, is anxious about performing nursing care interventions on a patient whose health is declining, and imagining performing these nursing care interventions under the guidance of the CCOS will probably help the nurse enjoy achieving the task.

Natural reward strategies aid persons to focus on the intrinsically motivating features of a given task and involve building more pleasurable features into a given task or refocusing attention away from less enjoyable features (Neck & Houghton 2006), which results in increased feelings of competence, self-control and a sense of purpose (Ryan & Deci 2017); for example, when a nurse is doing a routine task such as measuring a patient’s vital data, she or he may focus on the importance of doing a patient’s vital data.

For CCOS to be effective, clear leadership, constant education and training of bedside nurses are needed (Olsen et al. 2019). The motivation for developing the conceptual framework was to provide the authors with a rationale for the exploration and description of nurses’ opinions on self-leadership in a CCOS in a private hospital to facilitate the development of self-leadership for nurses in a CCOS.

## Research methodology

A qualitative, exploratory, descriptive and contextual design was followed to describe nurses’ experiences on their self-leadership in a CCOS. The research was conducted at a hospital in South Africa, which was one of 47 hospitals owned by a private hospital group. The hospital has six critical care units, two high-care units and 11 low-acuity wards, which include the following disciplines: medical, obstetrics, surgical, gastro-enterology, oncology and paediatrics. The sample was purposefully drawn from all nurses working in low-acuity wards. Bedside nurses (all nurse categories as defined by the South African Nursing Council [SANC]) who were directly involved in providing nursing care to patients in low-acuity wards were included in the sample. Non-permanent appointed nurses, nurses working in high-acuity wards and the CCOT were excluded from the sample. As seen in Table 1, eight focus group discussions were conducted with nurses. All nurse categories were included in the data collection, as bedside nurses’ experiences regarding their self-leadership were explored and not the scope of their practice. Most of the participants were known to the first author; therefore, an independent moderator who had experience in facilitating focus groups conducted the focus groups. Data saturation occurred after the eight discussions when no new data emerged (Ravitch & Carl 2016).

**TABLE 1:** Focus groups.

Focus group	Nurse category	Participants total
Focus group 1	Professional nurses	5
Focus group 2	Staff nurses	6
Focus group 3	Auxiliary nurses	6
Focus group 4	Auxiliary nurses	5
Focus group 5	Professional nurses	6
Focus group 6	Staff nurses	7
Focus group 7	Staff nurses	9
Focus group 8	Auxiliary nurses	6

*Source*: Prinsloo, C., 2018, ‘Self-leadership strategies of nurses in an outreach service at a private hospital group in Gauteng’, PhD thesis, School of Nursing, University of the Western Cape, Cape Town

Data analysis was performed utilising ATLAS.ti, a data analysis software programme and a computer-assisted ‘noticing things, collecting these things and thinking about them’ (NCT) analysis approach (Friese 2019). The first author scrutinised the data by noticing interesting things about the data, making notes and attaching preliminary codes. Collecting these things was performed by reading further and noticing similar items. Thinking about these observed and coded things led to finding patterns and relations for themes and categories that described the data. Table 2 provides an overview of the themes, categories and subcategories that were identified after analysing the data from the focus group discussions.

**TABLE 2:** Summary of the categories and subcategories identified from the data regarding the experiences of nurses’ self-leadership in the outreach service.

Theme	Category	Subcategory
1. Mindfulness of self-leadership through emerging of self-direction and self-motivation in the CCOS	Self-motivation of a CCOS	Requesting the CCOS
		Taking charge or assessing the patient
		Self-motivated to act as an advocate for patients during doctor rounds
	Role models for colleagues	Taking the lead when in charge of calling outreach experts
		Staff involvement in communication
	Training (mentoring and teaching) in self-leadership	
2. Acknowledgment of the role of the nurse in the CCOS as part of a team to obtain quality patient care	An outreach service as an essential service in delivering care to at-risk patients	Viewing positive outcomes or quality of patient care and patient satisfaction as essential
		Knowledge about patients’ conditions
	Assistance, support and guidance from patient outreach service team (agents)	Management of modified early warning system (MEWS)
		Support for nurses (recipients) asking for assistance
		Teamwork as a critical component of health care
	Challenges in calling the CCOS experts (agents)	The role of ward nurses as part of the team
		A need for outreach experts to facilitate a positive outcome for a deteriorating patient
3. Power of self-affirmation whilst delivering nursing care to a patient	Staff’s (recipients) sense of being affirmed for contributions towards the wellness of patients (terminus)	Self-confidence
		Being appreciated at work
		Passion for nursing and caring

*Source*: Prinsloo, C., 2018, ‘Self-leadership strategies of nurses in an outreach service at a private hospital group in Gauteng’, PhD thesis, School of Nursing, University of the Western Cape, Cape Town

CCOS, critical care outreach service.

### Trustworthiness

Leavy (2017:653), identified the following terms to describe techniques supporting trustworthiness: credibility, dependability, confirmability and transferability. The first author observed, made field notes and recorded the focus group discussions to assure credibility and confirmed with participants that the data accurately represented the information provided during focus group discussions. Dependability was achieved through the availability of an audit trail created whilst conducting the research by compiling and recording the research itself and the thoughts and decisions made during the research. Conformability was ensured through consensus discussion with anindependent experienced coder who confirmed the themes and categories that emerged from the data analysis. The research participants and the research setting were described meticulously to allow othersthe benefit of using the evidence (transferability).

### Ethical considerations

Ethical clearance was obtained from the Senate Research Committee of the University of the Western Cape (reference number: 12/7/6), whilst approval was obtained from the private hospital group (Netcare) (reference number: UNIV-2013-0007B), the hospital manager and written informed consent from participants. The names of the participants did not appear on the transcripts and participants could withdraw at any stage of the research process. No respondent had to be referred to a nearby prearranged psychologist, as the focus group discussions entailed minimal risk.

### Development of the conceptual framework for self-leadership of nurses in a critical care outreach service

The conceptual framework was developed based on practice-orientedtheory (Dickoff, James & Wiedenbach 1968). The first step in developing the conceptual framework was to explore nurses’ self-leadership in the CCOS. In the second step, the themes, categories and conclusions were evaluated to classify key attributes, characteristics and roles which formed the concepts that were then organised according to their features, as seen in Table 3.

**TABLE 3:** Outline of second step.

Category	Subcategory	Conclusions	Essential concepts in the conceptual framework
**Theme 1: Mindfulness of self-leadership through emerging of self-direction and self-motivation in the CCOS**			
Self-motivation of a CCOS	Requesting the CCOS	Nurses need to use the MEWS as a cue for assessing patients.	Self-cueing
		Nurses need to focus on their self-control and authority to plan the nursing care for their patients and to call the nurse expert when needed.	Create feelings of self-determination
	Taking charge/assessing the patient	Competent nurses need to have the knowledge and clinical observation skills to monitor patients’ vital data and calculate the MEWS.	Self-awareness
		Nurses have a sense of responsibility to look after patient.	Create feelings of self-efficacy
		Nurses need to take ownership, nursing their patients; they need to be proactive in following the MEWS guidelines.	Create feelings of self-determination
	Self-motivated to act as advocate for patients during doctors’ rounds	Nurses need to use self-talk to focus on what they need to do after measuring a patient’s vital data.	Examine thought patterns
		Nurses need to share their knowledge with other nurses with the purpose of assisting one another in the nursing care of deteriorating patients.	Building pleasant and enjoyable features into given activity
Role models for colleagues	Taking the lead when in charge of calling outreach experts	Planning is needed to address patients’ needs.	Examine thought patterns
		Experienced nurses visualise the activities needed to do when assisting a patient.	Identify and replace dysfunctional beliefs
		Nurses need to be knowledgeable about MEWS.	Self-awareness
		Nurses need to set goals to execute their duties excellently and diligently.	Self-goal setting
		Nurses need to have self-control to assess a deteriorating patient personally.	Self-observation
		Knowledge about their own capabilities is needed when nurses care for a deteriorating patient.	Self-assessment
		Nurses need to be proactive when attending to patients with an elevated MEWS.	Self-goal setting
		Self-observation amongst nurses is needed to determine their need for more knowledge to nurse a deteriorating patient.	Self-observation
		Nurses need to be committed to their patients and show interest in their patient.	Building pleasant and enjoyable features into a given activity
	Staff involvement in communication	Communication skills is needed amongst nurses to inform them what is expected from them.	Self-assessment
		Nurses need to focus being role models when they work in teams to care for a deteriorating patient.	Create feelings of self-determination
Training (mentoring, and teaching) in self-leadership		Nurses need to share their knowledge to empower their colleagues.	Create feelings of self-efficacy
		Self-observation is needed to determine nurses’ knowledge in the nursing care of their patients.	Self-observation
		Nurses need to be knowledgeable about MEWS.	Self-assessment
		Nurses need to attend skills developing training to develop their ability to nurse patients.	Self-assessment
**Theme 2: Acknowledgement of role of the nurse in the CCOS as part of a team to obtain quality patient care**			
An outreach service as an essential service in delivering care to at-risk patients	Viewing positive outcomes, quality of patient care and patient satisfaction as essential	Nurses need to be motivated to care for their patient.	Self-goal setting
		Nurses need to be confident in their knowledge that CCOS will assist them in dealing with a deteriorating patient.	Create feelings of self-efficacy
		Nurses need to share the vision that CCOS improve patients’ lives.	Identify and replace dysfunctional beliefs
		Nurses need to behave correctly working in teams when dealing with a deteriorating patient.	Self-goal setting
	Knowledge about patients’ conditions	Nurses need insight into the MEWS to ensure patients receive appropriate care.	Self-perception
		Nurses need to be experienced to deliver timely quality care to patients.	Create feelings of self-determination
		Nurses need to be knowledgeable about their patients.	Self-assessment
Assistance and support and guidance from patient outreach service team	Management of modified early warning system (MEWS)	Insight into the purpose of measuring a patients’ vital data by nurses is needed.	Examine thought patterns
		Nurses need to be enthusiastic to empower themselves.	Building pleasant and enjoyable features into tasks
		Nurses need to take responsibility with their knowledge and be accountable for delivering care to their patients.	Create feelings of self-determination
		Awareness should be demonstrated on calculating MEWS, making nursing care of their patients easier.	Shaping perceptions by focusing attention away from unpleasant aspects
		The MEWS chart should be known and guide nurses in self-determination of their actions.	Self-cueing
	Support for nurses asking for assistance	Communication channels should be available for nurses to call the outreach expert in case of an elevated MEWS.	Self-goal setting
		Nurses need to exercise self-control when delivering care for their patients and call the outreach expert when needed.	Self-goal setting
		Nurses need to be aware that CCOS contributes to the quality care delivered to patients.	Shaping perceptions by focusing attention away from the unpleasant aspects
	Teamwork as a critical component of health care	Self-confidence should be demonstrated in supporting their peers.	Create feeling of self-efficacy
**Theme 2: Acknowledgement of role of the nurse in the CCOS as part of a team to obtain quality patient care**			
Challenges in calling the patient outreach experts	The role of ward nurses as part of the team.	Nurses need to focus having a positive attitude to call the outreach expert.	Self-goal setting
	A need for outreach experts to facilitate a positive outcome for a deteriorating patient	Nurses need to regard the CCOS as a safety net when nursing patients.	Identify and replace dysfunctional beliefs and assumptions
**Theme 3: Power of self-affirmation whilst delivering nursing care to a patient**			
Staff’s sense of being affirmed for contributions towards the wellness of patients	Self-confidence	Nurses’ confidence increased when they managed a deteriorating patient in collaboration with CCOS.	Self-reward
	Being appreciated at work	Nurses felt proud when they have done something that saved a patient’s life.	Self-reward
		Nurses felt content when a patient was grateful to them.	Self-reward
	Passion for nursing and caring	Nurses need to focus on behaving in a proper manner when they are certain that their endeavours are going to result in positive outcomes.	Self-goal setting

*Source*: Prinsloo, C., 2018, ‘Self-leadership strategies of nurses in an outreach service at a private hospital group in Gauteng’, PhD thesis, School of Nursing, University of the Western Cape, Cape Town

CCOS, critical care outreach service.

After the first author identified the main attributes, characteristics, assumptions, themes and categories, the concepts were organised and categorised according to the five components in the practice-oriented theory survey list (Dickoff et al. 1968), which was the ‘reasoning map’ for developing and constructing the conceptual framework (third step). These five components are framework, agent, recipient, procedure, dynamics and terminus. The following questions clarified and created an understanding of the concepts:

- In what context is the activity (CCOS) performed (framework)?
- Who performs the activity (agent)?
- Who is the recipient of the activity (recipient)?
- What is the energy source for the activity (dynamics)?
- What is the guiding procedure, technique or protocol of the activity (procedure)?
- What is the endpoint of the activity (terminus)? (Dickoff et al. 1968)

These questions led to the creation of the conceptual framework for the self-leadership of nurses in the outreach service of a private hospital in Pretoria. Table 4 displays how these questions were answered in the context of this study.

**TABLE 4:** Reasoning map.

Component	Concept clarification
Framework	The private hospital group applying CCOS in general wards.
Agent	The critical outreach nurse expert is a professional nurse with critical nurse specialist competencies for advanced practice nurses.
Recipient	The primary recipients are bedside nurses working in low-acuity wards that are part of the CCOS. The secondary recipient is the patient in a low-acuity ward whose condition is at risk of declining or is already declining.
Dynamics	The underlying dynamics in self-leadership of a CCOS are the training of nurses and mindfulness.
Procedure	The agent inspires the primary recipient to apply self-leadership activities in the CCOS to manage the secondary recipient’s well-being.
Terminus	The accomplishment of the activities (procedure) or end results in nurses applying self-leadership in the CCOS.

*Source*: Prinsloo, C., 2018, ‘Self-leadership strategies of nurses in an outreach service at a private hospital group in Gauteng’, PhD thesis, School of Nursing, University of the Western Cape, Cape Town

CCOS, critical care outreach service.

#### In what context is the activity performed (framework)?

In 2005, a nurse-led CCOS was introduced in South Africa at a private hospital in Pretoria, which was the context in which the activity was performed. This private hospital emergency department is endorsed as a level 2 trauma unit and has six critical care units, 18 theatres (for cardiothoracic, nephrology, orthopaedic, neurology and general surgery) and 11 low-acuity wards (for oncology, medical, surgical, orthopaedic obstetrics and paediatric care) that total to approximately 500 beds.

The CCOS is essential for assisting bedside nurses (all nurse categories) in providing individualised nursing care for patients. Wood et al. (2017) indicated that CCOS enhances nurses’ skills and competence, which leads to an increase in their confidence.

In this private hospital, the CCOT was predominantly nurse-led and involved a team of nurses, namely the critical care outreach nurse expert and bedside nurses working in low-acuity wards. The patient medical practitioner also forms part of the CCOT. Patients at risk of deteriorating are recognised by nurses who measure the patients’ vital data and determine the patients’ MEWS. The MEWS is a set of criteria to identify early deterioration in patients. If a patient is identified as a patient whose health is at risk of declining, the outreach nurse expert is called to assess the patient. The outreach nurse expert will assist the bedside nurse with nursing interventions needed and monitor the patient. The patient medical practitioner is also informed of interventions implemented and that the CCOS has been activated to monitor the patient. The medical practitioner may request additional interventions to be implemented, and the outreach expert nurse has the competency to evaluate the outcome of the interventions implemented. The outreach nurse expert enhances the bedside nursing skills by explaining to the bedside nurse to monitor for specific outcomes that are expected from interventions implemented, for example, to monitor if a patient’s breathing improves after pain medication is given to a patient who is in pain and displays signs of hyperventilation. Critical care outreach service patients are monitored more frequently than patients not identified as at-risk patients. Better nursing and patient outcomes are associated with work milieus that are supportive of professional practice (Stein-Parbury 2017). Empowerment of nurses increases feelings of competence, and being responsible for monitoring patients whose health is at risk of declining provides a sense of self-control and purpose which is naturally rewarding to nurses (Ryan & Deci 2017).

#### Who performs the activity (agent)?

The agent is the critical care nurse expert, that is, a critical nurse specialist with competencies for advanced practice nurses developed by SANC (2014), registered at the SANC. These nurses are self-directed critical care specialised nurses with the goal of promoting the health of patients (secondary recipients) in assisting bedside nurses (primary recipients) with nursing activities for patients whose condition is at risk of declining or declining. Hansen-Turton, Sherman and King (2015) defined nursing expertise as the use of all features of practising evidence-based nursing in solving health care problems. Critical care outreach expert clinical expertise enables nurses to holistically assess patients, and they have the skills and knowledge to assist bedside nurses with timeous nursing care interventions for a patient whose health is at risk of declining or declining at the moment.

The critical care nurse expert as a mentor empowers the bedside nurse and is in a position of authority when practising self-direction when they take the lead and provide assistance and support to bedside nurses with nursing care interventions to promote patients’ health. The American Association of Colleges of Nursing defines mentoring as:

A formalised practice whereby a more knowledgeable and experienced person acts in a supportive role of supervising and inspiring reflection and learning within a less experienced and knowledgeable person, to facilitate that person’s career and personal development. (DeWitty, Choi & Zachary 2017:5)

The critical care nurse experts as agents need to reflect on their practice, displaying self-awareness (Bang & Reio Jr 2017), and should indicate to bedside nurses that they want to support and guide them with nursing care activities to improve patients’ health whose health is at risk of declining.

The agent as a team leader is a critical care nurse expert who leads and directs the functionality of the CCOT. As a team leader, the critical care outreach nurse expert is competent in making decisions on which activities are needed to improve a patients’ health status. Dupree (2018) stated that the role of team leaders is to guide team members and provide them with direction and support so that they can complete the assigned goal successfully. The self-motivated critical care outreach nurse experts empower bedside nurses by employing self-direction when they guide and support these nurses with the nursing care interventions for patients whose health is at risk of declining or declining at the moment. In self-direction, the critical care nurse expert as the agent directs their own thoughts, approaches and behaviours to modify their behaviour in order to gain the desirable outcomes (Watson & Tharp 2014). The support that bedside nurses receive from the critical care outreach nurseexpert transforms the nursing care provided to patients into a more positive experience (Cherry 2018), which makes nursing care more naturally rewarding.

Neck et al. (2020) indicated that self-leadership stems from intrinsic motivation theory and more specifically self-determination theory. The critical care outreach nurse experts render self-leadership by being confident about their skills and knowledge when attending to a patient whose condition is at risk of declining or declining at that moment. Self-efficacy is the belief that one has the essential resources, namely skills and knowledge, to meet the needs of one’s specific goal (behaviour-focused action) (Ayub, Kokkalis, & Hassan 2017).

#### Who is the recipient of the activity?

Two recipients were identified; namely, the bedside nurse who works in low-acuity wards and who is part of the CCOS is the primary recipient, and the secondary recipient is the patient in a low-acuity ward whose health is at risk of declining or declining at the moment. The bedside nurses include all nurse categories (professional, staff and auxiliary nurses) as stipulated in the *South African Nursing Act 33* (South Africa 2005) and are thoughtful therapeutic practitioners, who exhibit competencies, principles, attitudes, clinical skills and expertise in the endorsement of patients’ health, for example, by observing the patients’ vital data. The bedside nurse (primary recipient) as a team member of the CCOS must demonstrate self-leadership through these practices: self-motivation, self-direction, being a role model, recognition of their own role in the CCOS as a vital service in nursing, concerning patient outcomes, team assistance and challenges and self-affirmation.

The bedside nurses need self-motivation to support and lead their self-leadership in the CCOS. Messineo, Allegra and Seta (2019) stated that self-motivation occurs when nurses follow a pupose, which creates emotions of satisfaction in the activity. Nurses are self-motivated to act when they have to chance to independently decide on meaningful interventions that allow them to use their competencies. The bedside nurses should exhibit their awareness of their responsibility through behaviour-focused strategies when attending to the patient, utilising appropriate behaviour which will contribute to delivering of quality nursing care to the patient as the secondary recipient. Setting goals for themselves, bedside nurses motivate themselves to grow to be the best at what they do or at least progress in their performance. To achieve this, the bedside nurses need a proactive approach and self-control to care for patients whose health might decline or is declining. Bedside nurses practice self-leadership cognitively through self-talk (constructive thought process) on what nursing care activities are needed for the patient whose health is declining. The bedside nurse applies natural reward strategies by implementing self-control (autonomy) when the bedside nurse becomes aware that the patient’s health is declining, reports it to a more senior nurse and demonstrates their ownership of the situation by following the CCOS guidelines when a patient’s health is declining. Bedside nurses should be competent to provide standard nursing care and to show a positive feeling of self-efficacy (Ayub et al. 2017).

The bedside nurse must be mindful of their own self-leadership and need to be a role model for their colleagues to encourage each other in the CCOS. Being role models, nurses set goals and perform their duties excellently and persistently by setting high standards when providing nursing care to patients. Nurses should develop realistic expectations of their own capabilities whilst theyprovide nursing care to a patient whose health is declining. This can be performed through self-assessment of a behaviour-focused activity where they need to determine their need to improve their knowledge about some aspects of nursing patients whose health is declining. They demonstrate self-control and self-direction when the bedside nurse reports a patient’s declining health to a more senior nurse. As role models, nurses need to develop their knowledge and skills and show their commitment to improving their patients’ (secondary recipient) health by attending training. They should be hands-on and exhibit their competence when attending to patients whose health is declining. Being competent in attending to patients creates feelings that are naturally rewarding. Neck et al. (2020) indicated that natural reward strategies are self-motivating when a task is enjoyable, and that leads to increased feelings of competence. They should share their expertise, involving their colleagues through communicating to them the expected nursing interventions needed to provide nursing care to the patient. Nurses also use constructive thought patterns when they visualise the successful completion of nursing interventions. This involves substituting dysfunctional beliefs or assumptions that could hamper the treatment of thepatient with visualising and performing the nursing interventions under the guidance of the outreach nurse expert and replacing the assumptions that they are not competent to perform these interventions. Passi and Johnson (2016) stated that role modelling is a forceful process where onlookers, namely bedside nurses, intentionally and subconsciously decide if the observed behaviour will be applied to their own behaviour. Furthermore Jochemsen-Van der Leeuw, Wieringa-De Waard and Van Dijk (2015) mentioned that when a person is aware that he or she is a role model, it improves role model behaviour.

Nurses acknowledge their own role through self-awareness as vital concerning patient outcomes and activating the outreach nurse expert. Nurses should acknowledge their role as team members of the CCOS and need to motivate themselves to provide the appropriate care for a patient, which can be seen as the goal for a patient whose health is declining. They ought to exercise their understanding of the MEWS, which can be a cue to act correctlyto make sure that the patient receives the correct nursing care to remain stable. Zuckerman, Friedman and Castro (2018:17) are of the view that self-awareness refers to self-examination: understanding of one’s own choices, motivation and behaviour. When nurses have the knowledge to attend to patient nursing care needs, they display their confidence, which is a natural reward and demonstrates their self-control when they attend to these nursing care needs such as calling the outreach nurse expert if needed. The availability of the CCOS and the awareness that they (primary recipient) can call the outreach nurse expert (agent) to assist in caring for a declining patient (secondary recipient) contributes to providing quality nursing care to the patient and is naturally rewarding for nurses.

Through self-observation, nurses should be aware of the skills and knowledge that need to be improved, and their self-leadership will be demonstrated when they empower themselves by improving their skills and knowledge. Empowering themselves influences their behaviour and affirms nurses that they contribute to providing quality nursing care to patients. According to Armitage and Rowe (2017:490), self-affirmation increases motivation and changes behaviour. A nurse might adjust his or her behaviour to comply with professional standards when she or he is confident that his or her effort to change his or her behaviour will result in positive patient outcomes. Patients who express their gratitude to nurses for the nursing care they received contribute to nurses’ self-affirmation, creating feelings of competence and self-confidence which are self-rewarding.

#### What is the energy source for the activity (dynamics)?

The data analysis revealed that mindfulness is an underlying energy source for self-leadership. In addition, Tutzer and Sachse (2018:353) defined mindfulness as the intentional and non-judgemental self-observation of incidents involving self-regulation of perception and orientation towards incidents in the current moment. Mindfulness enables nurses to focus on what is necessary to do at that instant and focus their behaviour towards their self-leadership. The goal of self-assessment amongst nurses is to develop self-awareness that guides themto perform basic professional actions, namely providing nursing care to patients whose health is declining. Yu and Zellmer-Bruhn (2018:340) stated that team members (such as in the CCOS) require mindfulness to form mutual insights on their interactions and increase those which are valuable for the team. Nurses need to focus on listening mindfully to their patients and the CCOT, which is needed when providing nursing care for a patient whose health is declining. Mindful listening is beneficial for positive communication and can create an internal calm in team members, releasing them from presumptions and biases, which will to a great extent support the team leader (such as the outreach nurse expert) (Ackerman 2017).

Another underlying energy source that was revealed through the data analysis is training. Training focuses on building knowledge, skills that are directly related to work and are planned to increase the efficiency of employees (Warren et al. 2018:117). Health care organisations can empower nurses through training programmes to increase their knowledge and competence. Through self-awareness, nurses are aware of skills and knowledge that need to be improved and will attend these training programmes. Patients whose health is declining in general wards provide challenges for the bedside nurse; therefore, training is needed to improve nurses’ competence. The outreach nurse expert empowers (informal training) bedside nurses when they assist bedside nurses with guidance and advice on the nursing care needed for their patients. Bedside nurses’ (recipient) knowledge increases, and they develop skills that increase their confidence and competency. Van Vianen et al. (2018:594) stated that mentoring is an important part of training and preparing bedside nurses to deal with challenging job demands. The mentor (outreach nurse expert) is at the core of such training, and mentoring bedside nurses provides them with the prospect to grow, which creates job satisfaction and enhances bedside nurses’ competencies (Goodyear & Goodyear 2018). Further, Bang and Reio Jr (2017:150) reported that mentoring contributes to self-efficacy. Mentoring bedside nurses as a training method in the CCOS increases nurses’ competency and self-efficacy and encourages job satisfaction. The underlying dynamics of mindfulness and training will encourage self-leadership amongst bedside nurses in the CCOS.

#### What are the guiding procedures for self-leadership of bedside nurses in a critical care outreach service?

Dickoff et al. (1968:423) stated that the guiding procedures are the blueprint for the execution of an activity. In the CCOS, the mobilisation of self-leadership is needed to provide appropriate nursing care for patients whose health is declining. Bedside nurses utilising self-leadership in a CCOS should apply the following activities to lead and guide themselves in providing nursing care to patients, namely self-motivation, role modelling, teamwork and self-affirmation.

Neck et al. (2020) stated that the self-determination theory suggests that enhancing self-motivation, competence and self-determination are needed. The ability to motivate yourself to provide quality nursing care is crucial in self-leadership. Self-motivation is the spontaneous feeling of attraction and delight in an activity that supplies rewards (Deci, Olafsen & Ryan 2017:21). Duckworth et al. (2019) defined self-control as the self-initiated management of conceptions, outlooks and actions when enduringly valued goals conflict with briefly more rewarding goals. Self-motivation allows a person through personal self-control (mindfulness) to take action to render the needed effort to carry out a task such as providing nursing care to patients. Nurses motivate themselves through self-control to measure patients’ vital data and follow the MEWS guidelines to call the outreach nurse expert if needed. Cherry (2018) stated that self-determination theory suggests that people are motivated to develop and transform through psychological needs such as the need to be competent in what they do. Self-directed behaviour increases feelings of competence when nurses motivate themselves to learn more.

According to Price-Mitchell (2017), a role model functions as an example of motivating people with integrity, determination and compassion. In the health care organisation, nurses (recipients) act as role models when they demonstrate an interest in their patients’ health and accept responsibility for their patients’ nursing care. They exercise self-control and change their behaviour if needed to be proactive in providing quality nursing care to their patients. Nurses are proactive when they act without delay to ensure patients receive the appropriate nursing care when they focus on visualising what to do to assist a patient whose health is declining. Nurses also employ constructive thought patterns through planning and visualising the successful completion of tasks to address the needs of patients. Nurses as role models for their peers increase the likelihood that peers (other nurses) could change their behaviour to meet task demands and face new challenges positively (Kranabetter & Niessen 2017). Nurses as role models should through self-assessment know the boundaries of their knowledge and skills, have realistic expectations of their competencies and should call for outreach expert nurses when needed.

Heathfield (2018) stated that to create teamwork, a work culture that values collaboration between employees need to be created and that employees understand and believe that reasoning, creating plans, making decisions and activities have better outcomes when performed together. Jooste (2017) stated successful teams and team members should have an understanding of the team goal. Behaviour-focused self-leadership involves the setting of goals (Neck et al. 2020) and forms an important part of successful self-leadership. Bedside nurses working as a team set goals such as calling the outreach nurse expert for assistance when a patient’s health is declining. Being a member of a team is an important aspect of bedside nurses’ work, which helps to enhance the effectiveness of quality nursing care (ed. Peteva 2017). Team members provide support and empower other team members, which create feelings of competence and unity amongst the team members. Feelings of competence are naturally rewarding, which is an approach applied in self-leadership (Neck et al. 2020).

Bedside nurses’ positive attitudes are imperative in employing self-leadership approaches. Nurses feel proud when they have provided quality nursing care, and patients express their appreciation to nurses, which is an opportunity for self-affirmation. White (2015) emphasised the importance of employees needing to know what they do matters. This creates feelings of self-affirmation for nurses.

#### What is the endpoint of the activity (terminus)?

The final activity of the framework is the accomplishment of activities and procedures bedside nurses use in applying their self-leadership in the CCOS, which is the core outcome. The CCOS offers the critical care skills and knowledge of the outreach nurse expert to patients whose health is declining in low-acuity wards.

### Strengths and limitations of the study

The conceptual framework provided the authors with an understanding of how nurses’ self-leadership affects the application of CCOS. One limitation is that the study was limited to one private hospital, and no other hospitals were included in the research. Despite these limitations, the developed conceptual framework is significant, as according to the authors, no conceptual framework has been published in this context.

## Conclusion

The conceptual framework can assist managers in the development of training programmes to improve nurses’ self-leadership and ultimately improve their competence and provide qualty nursing care to patients. The development of the conceptual framework was part of another study. The conceptual framework, based on the five components of the practice-orientated theory by Dickoff, was adapted to provide the authors with a rationale for the development of self-leadership strategies for nurses to be implemented in a CCOS.
